# *Fusobacterium nucleatum*: An Overview of Evidence, Demi-Decadal Trends, and Its Role in Adverse Pregnancy Outcomes and Various Gynecological Diseases, including Cancers

**DOI:** 10.3390/cells13080717

**Published:** 2024-04-20

**Authors:** Arunita Ghosh, Ken Jaaback, Angela Boulton, Michelle Wong-Brown, Steve Raymond, Partha Dutta, Nikola A. Bowden, Arnab Ghosh

**Affiliations:** 1 School of Biomedical Sciences and Pharmacy, University of Newcastle, Callaghan, NSW 2308, Australia; arunita.ghosh@newcastle.edu.au; 2 Drug Repurposing and Medicines Research Program, Hunter Medical Research Institute, New Lambton Heights, NSW 2305, Australia; michelle.wong-brown@newcastle.edu.au; 3 Hunter New England Centre for Gynecological Cancer, John Hunter Hospital, Newcastle, NSW 2305, Australia; kenneth.jaaback@health.nsw.gov.au; 4 School of Medicine and Public Health, University of Newcastle, Callaghan, NSW 2308, Australia; 5 Newcastle Private Hospital, Newcastle, NSW 2305, Australia; angeboulton@gmail.com (A.B.); steve@steveraymond.com.au (S.R.); 6 Department of Medicine, Division of Cardiology, University of Pittsburgh, Pittsburgh, PA 15261, USA; duttapa@pitt.edu; 7 Department of Immunology, University of Pittsburgh, Pittsburgh, PA 15261, USA

**Keywords:** adverse pregnancy outcomes, dysbiosis, *Fusobacterium nucleatum*, gynecological cancers, gynecological diseases, opportunistic pathogen, immunomodulation

## Abstract

Gynecological and obstetric infectious diseases are crucial to women’s health. There is growing evidence that links the presence of *Fusobacterium nucleatum* (*F. nucleatum*), an anaerobic oral commensal and potential periodontal pathogen, to the development and progression of various human diseases, including cancers. While the role of this opportunistic oral pathogen has been extensively studied in colorectal cancer in recent years, research on its epidemiological evidence and mechanistic link to gynecological diseases (GDs) is still ongoing. Thus, the present review, which is the first of its kind, aims to undertake a comprehensive and critical reappraisal of *F. nucleatum*, including the genetics and mechanistic role in promoting adverse pregnancy outcomes (APOs) and various GDs, including cancers. Additionally, this review discusses new conceptual advances that link the immunomodulatory role of *F. nucleatum* to the development and progression of breast, ovarian, endometrial, and cervical carcinomas through the activation of various direct and indirect signaling pathways. However, further studies are needed to explore and elucidate the highly dynamic process of host–*F. nucleatum* interactions and discover new pathways, which will pave the way for the development of better preventive and therapeutic strategies against this pathobiont.

## 1. Introduction

The human microbiome is a diverse collection of microorganisms, such as bacteria, archaea, viruses, and eukaryotes, that exist both inside and outside of the human body. These microorganisms inhabit and interact with the human body by commensalism, mutualistic, or pathogenic behavior, impacting human health either beneficially or detrimentally by contributing to sound health or disease through the enhancement or impairment of metabolic and immune functions [1,2].

Among the trillions of microorganisms present in the human body, bacteria are by far the most predominant [3,4]. Studies in recent decades have delineated the structure and the functional capacity of the bacterial microbiome in both the healthy state and a variety of disease states [2,5]. In healthy individuals, pathogenic and symbiotic bacteria coexist without causing harm to the host. However, when there is a disturbance in this balance or a change in the host’s bacterial community due to factors such as infectious illnesses, specific diets, or the prolonged use of antibiotics or other bactericidal medications, dysbiosis can occur. This can cease the normal beneficial interactions, thereby increasing the host’s susceptibility to various infections, and the nature of those depends on the anatomical sites involved [2,6]. An article published in ‘The Lancet’ in 2022 [7] reported on a systematic analysis of the Global Burden of Disease Study 2019 and found that bacterial infections are the second-leading cause of death worldwide, responsible for one in eight of all global deaths. Pathogenic bacteria play a role in different inflammatory diseases, including cancers [8,9,10]. Additionally, emerging evidence [11,12,13,14,15,16] has demonstrated a clear role of intestinal and genital bacteria in the development of various pregnancy complications, gynecological diseases (GDs), and gynecological cancers (GCs). Among these bacteria, *Listeria monocytogens*, *Chlamydia trachomatis*, *Neisseria gonorrhoeae*, *Treponema pallidum*, *Trichomonas vaginalis*, *Escherichia coli*, *Shigella* sps, *Staphylococcus epidermidis*, *Porphyromonas somerae* and *Fusobacterium nucleatum* have been most prevalent in various gynecological complications over the last few years [16,17,18,19,20,21,22,23,24].

However, one bacterium that has received considerable attention in cancer microbiota studies is *Fusobacterium nucleatum* (*F. nucleatum*). This is due to its high abundance and relationship to poor prognosis in various types of cancers (colorectal, head and neck cancer, esophageal cancer, pancreatic cancer, and prostatic cancer), including GCs (cervical carcinoma, and breast cancer) [24,25,26]. Recent worldwide studies have also reported the association of *F. nucleatum* with pregnancy complications (chorioamnionitis, spontaneous abortion, preterm birth, stillbirth, neonatal sepsis, preeclampsia, etc.) [27,28,29]. Further, studies have also stated the association of *F. nucleatum* with various GDs, including polycystic ovarian syndrome (PCOS), endometriosis, and bacterial vaginosis (BV) [30,31,32], in addition to cancers.

An obligate anaerobic Gram-negative bacillus *F. nucleatum* belongs to the genus *Fusobacterium*, so-named based on its slender shape and spindle-like tips at both ends. It often exists as a commensal in various sites of the body, especially in the human oral cavity [33]. *F. nucleatum* is a heterogeneous species, with five known subspecies (i.e., *animalis*, *fusiforme*, *nucleatum*, *polymorphum*, and *vincentii*) [34]. Originally, an oral pathobiont, *F. nucleatum*, is known to coaggregate with various microbial species in the oral cavity, playing a pivotal role in dental plaque formation [16,24,35,36]. However, it has often been implicated in various extra-oral diseases, including cancers, due to its transmission via the hematogenous route [16,37] and by its virulence mechanisms, including the ability to induce aberrant inflammation and tumorigenesis [16]. *F. nucleatum*, an adhesive bacterium, acts as an opportunistic pathogen in patients with compromised health conditions, particularly when it invades sterile locations such as the root canal [25,35].

Various studies have provided epidemiological and/or experimental evidence that a significant association between *F. nucleatum* and colorectal cancers (CRCs) exists, also delineating its crucial role in the pathogenicity, development, and prognosis of CRC [38,39,40,41,42]. Additionally, both molecular mechanism studies and epidemiological evidence have shown a positive correlation between pre-existing inflammatory lesions, such as periodontitis, and an increased risk of cancer [43]. Extensive research studies with epidemiological evidence and mechanistic linkage involving *F. nucleatum* and various GDs are underway. The role of *F. nucleatum* in GDs, including GCs, is emerging and discloses the versatile ways in which this bacterium contributes to the development, growth, spread, and treatment response to these diseases.

**Figure 1:**
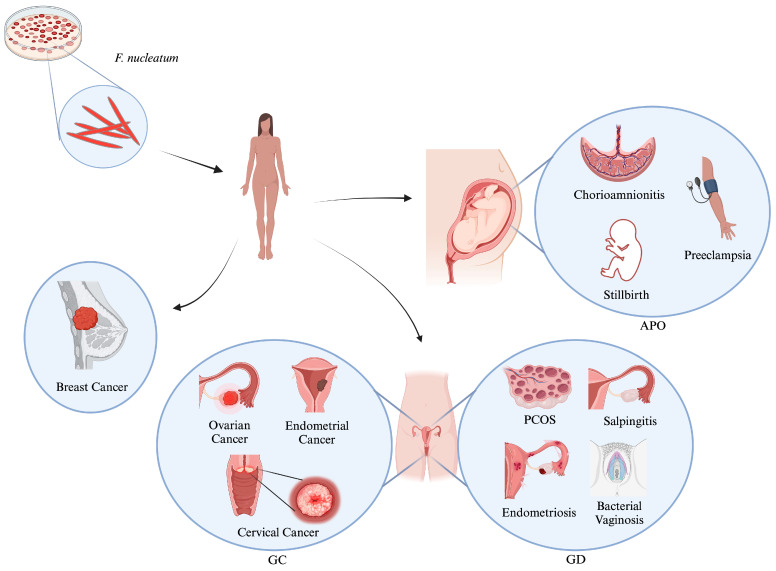
A schematic representation depicting the role of *F. nucleatum* in adverse pregnancy outcomes (APOs), gynecological diseases (GDs), and gynecological cancers (GCs).

This review aims to delve into recent evidence and future directions of *F. nucleatum*, particularly in gynecology, including their genetics and mechanistic role in promoting adverse pregnancy outcomes (APOs), various GDs, including cancers, and the challenges of developing diagnostics and therapeutics for *F. nucleatum*. This review focuses on the current evidence for *F. nucleatum* in disease pathogenesis and/or tumor development, highlighting the similarities and differences between *F. nucleatum*-associated GDs. We also explore the diverse facets of this bacterium’s interaction with the host with potential gynecological implications. Thus, this review provides a comprehensive understanding of the role of *F. nucleatum* in APOs and GDs, including cancers (Figure 1), and a potential rationale for future research studies based on *F. nucleatum* as a predictive biomarker and/or a target for anti-tumor therapy.

## 2. Evidence Acquisition and Synthesis

An extensive literature search was conducted using the electronic databases PubMed and Google Scholar from 2019 until 2023. The keywords used were *F. nucleatum* in association with various APOs (chorioamnionitis, neonatal deaths, preterm births, stillbirths, and spontaneous abortion), different GDs (polycystic ovary syndrome, salpingitis, endometriosis, and BV), and cancers (breast cancer, ovarian cancer, endometrial cancer, and cervical cancer). The retrieved papers were carefully chosen based on title and abstracts. Then, the full text of the selected papers was evaluated. The references of the reviews were manually searched to ensure no relevant references were missed. Full article texts, including literature reviews and chapters were incorporated for analysis relating to the review’s objectives. However, regarding the clinical terminology, the most relevant information was incorporated from online sources, irrespective of the article length and type. Only articles written in English were considered for this review, and preprints were excluded. The figures and graphical abstract were created with BioRender.com.

## 3. *F. nucleatum* in Adverse Pregnancy Outcomes

APO is a broad term comprising health complications that affect the mother, newborn baby, or both during pregnancy, labor, delivery, and the postpartum period [44]. These health complications vary from pregnancy to pregnancyand include chorioamnionitis, preterm birth, spontaneous abortions, stillbirth, neonatal sepsis, low birth weight, preeclampsia, and gestational diabetes mellitus (GDM) (please refer to Box 1) [16,23,34,45,46,47,48,49,50]. APOs are responsible for an enormous burden of maternal and infant mortality and morbidity worldwide [51,52].

Box 1 Adverse pregnancy outcomes 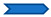 definition, etiologies, and symptoms.

**Chorioamnionitis**

Acute inflammation can occur in the chorion, amnion, or both of the extraplacental membranes or chorionic plate. This is known as the maternal inflammatory response (MIR). In some cases, there may also be acute inflammatory cell extravasation from the umbilical cord vasculature or chorionic plate vessels, known as the fetal inflammatory response (FIR). These responses can occur prior to, during, or after labor [53]. The inflammation is often caused by chronic, subacute, or acute infection, typically due to ascending polymicrobial bacterial infection after membrane rupture [54]. Symptoms may include fever, maternal and/or fetal tachycardia, uterine tenderness and inflammation, foul-smelling amniotic fluid, or an elevated white blood cell (WBC) count, which may lead to pregnancy complications [55].

 

**Preterm birth**

Any birth that occurs before 37 completed weeks of gestation. It is a major cause of neonatal mortality worldwide [56]. Various factors contribute to preterm birth, including stress, infection, placental abruption, placenta previa, substance use, history of abortion, inadequate prenatal care, smoking, maternal age (<18 or >40), poor nutrition, fetal anomaly, fetal growth restriction, oligohydramnios, polyhydramnios, vaginal bleeding, premature preterm rupture of membranes (PPROM), and environmental factors. Some common signs of preterm labor include regular contractions before the expected gestational age, cervical changes, pelvic pressure, menstrual-like cramps, watery vaginal discharge, and lower back pain [57].

 

**Spontaneous abortions**

Natural loss of pregnancy prior to twenty weeks of gestation [58], or with a fetus born weighing < 500 g [27]. It is estimated that about 50% of miscarriages are caused by fetal chromosomal abnormalities. Other contributing factors include advanced maternal age, alcohol consumption, smoking, cocaine use, and chronic diseases such as diabetes, celiac disease, and autoimmune conditions like anti-phospholipid antibody syndrome [58]. Rapid conception after delivery and infections such as cervicitis, vaginitis, HIV, syphilis, and malaria are also common risk factors. Symptoms often include abdominal and pelvic cramping, vaginal bleeding, and tachycardia. In cases of significant bleeding, patients may experience symptoms of hypovolemia, even without sepsis [58].

 

**Stillbirth**

The death of a fetus with a birth weight of 500 g, or if birth weight is unobtainable, gestational age of 22 weeks or a crown-to-heel length of 25 cm. According to the World Health Organization (WHO), all fetuses and infants weighing at least 500 g at birth should be included in statistics. However, for international reporting and comparisons, WHO also recognizes a higher limit (1000 g/28 weeks/35 cm) for third-trimester stillbirths [59]. This can be caused by various factors, such as intrapartum complications, hypertension, diabetes, infection, congenital and genetic abnormalities, and placental dysfunction. Certain risk factors, including advanced maternal age, teenage pregnancies, maternal nutritional status, infections, prior pregnancy losses, complicated pregnancies, and multiple pregnancies, can increase the likelihood of stillbirth. Symptoms may include a cessation of fetal movement and kicks, vaginal spotting or bleeding, and back pain for the mother [60].

 

**Neonatal sepsis**

An infection in the bloodstream in newborn infants less than 28 days old. This remains a leading cause of morbidity and mortality among neonates. It is divided into two groups, based on the time of presentation after birth: early-onset sepsis (EOS) and late-onset sepsis (LOS). EOS refers to sepsis in neonates at or before 72 h of life, while LOS occurs at or after 72 h of life. EOS is typically caused by the transmission of pathogens from the female genitourinary system to the newborn or fetus, while LOS is usually transmitted from the surrounding environment after delivery, such as through contact with healthcare workers or caregivers. Risk factors for this condition include advanced maternal age, chorioamnionitis, a weakened immune system, and the use of invasive devices. Symptoms and signs may include irritability, lethargy, and poor feeding, as well as respiratory distress, fever, hypothermia, hypotension, and shock. In some cases, hyperglycemia, hypoglycemia, acidosis, or hyperbilirubinemia may be the only indicators of the condition when other factors are absent [61].

 

**Preeclampsia**

A multisystem condition arising during pregnancy including hypertension and proteinuria. According to Karrar and Hong (2023) [62], the initial parameters for identifying preeclampsia are a systolic blood pressure of 140 mm Hg or higher, or a diastolic blood pressure of 90 mm Hg or higher on two separate occasions at least 4 h apart. Alternatively, a shorter interval timing of a systolic blood pressure of 160 mm Hg or higher or a diastolic blood pressure of 110 mm Hg or higher, can also indicate preeclampsia. These criteria must be met after 20 weeks of gestation. There are various potential causes of preeclampsia, including uteroplacental ischemia, maternal infection and inflammation, maternal intestinal dysbiosis, maternal obesity, sleep disorders, hydatidiform mole, fetal diseases, autoimmune disorders, placental aging, breakdown of maternal-fetal immune tolerance, and endocrine disorders [63]. Common signs and symptoms of preeclampsia include new onset headache, right upper quadrant or epigastric pain with associated nausea or vomiting, shortness of breath, and increased swelling [62].

 

**Gestational diabetes**

Any degree of glucose intolerance that occurs or is first recognized during pregnancy and typically resolves after the baby is born. It is recommended to screen for gestational diabetes between 24 and 28 weeks of pregnancy using a 50 g, 1 h oral glucose tolerance test. If the results are abnormal, with a value of 130 mg/dL (7.22 mmol/L) or higher, a confirmatory test is necessary using a 100 g, 3 h oral glucose tolerance test. The following values are considered abnormal: a value over 180 mg/dL in the first hour, over 155 mg/dL in the second hour, and over 140 mg/dL in the third hour. A diagnosis of gestational diabetes is established if two or more of these values are abnormal [64]. However, according to the Australian Diabetes in Pregnancy Society (ADIPS), a single abnormal result of either fasting > or = to 5.1 mmol/L or 1 h > or = to 10.0 mmol/L or 2 h > or = to 8.5 mmol/L is considered diagnostic for gestational diabetes. The etiology is thought to be related to dysfunction of the pancreatic beta cells and/or delayed response of the beta cells to changes in blood sugar levels, as well as increased insulin resistance due to hormones released by the placenta. Contributing factors may include a body mass index (BMI) over 25, decreased physical activity, a first-degree relative with diabetes mellitus, a history of gestational diabetes or a previous newborn with macrosomia, and metabolic comorbidities such as hypertension, low HDL, triglycerides over 250, polycystic ovarian syndrome, an abnormal oral glucose tolerance test, acanthosis nigricans, or a medical history of cardiovascular diseases. Clinical signs may include disproportionate weight gain, obesity, an elevated BMI, and fatigue during pregnancy [64].

*F. nucleatum*, although often found in healthy human placentas [65], has by far been the most prevalent bacterium implicated in various APOs in the last 5 years [16,29,66,67]. Chorioamnionitis and preterm birth, the common complications of pregnancy causingsignificant maternal, perinatal, and long-term adverse outcomes, have often been associated with a high abundance of *F. nucleatum* in the placenta, amniotic fluid and fetal membranes [15,16,25,28,48,50,65,68,69,70,71,72,73,74]. A recent case report from Italy by Bonasoni et al. [75] has shown that *F. nucleatum* was present in the placental membrane of a 26-year-old woman who presented at the hospital with painful uterine contractions at 23 weeks gestation. She delivered a preterm infant with premature rupture of membranes, but unfortunately, the baby girl died soon after birth. The placental histopathology revealed severe necrotizing chorioamnionitis and funisitis, with Fusiform bacteria consistent with *F. nucleatum*. The neonatal autopsy showed organ congestion and mild pericardial, pleural, and abdominal serous effusions, but no congenital anomalies were found. Histological analysis showed an abundance of neutrophils in the lungs and scattered granulocytes in the gastric lumen and large bowel. The fetal inflammatory response was evident with acute funisitis, chorionic vasculitis, and acute deciduitis. Microbiologically, *F. nucleatum* was only isolated from the infant’s lung. It is worth noting that despite -severe inflammation, the mother did not experience any symptoms (usually, *F. nucleatum* chorioamnionitis presents with a raised temperature). However, the mother presented with mild and diffuse gingivitis and had undergone a dental procedure one day prior to delivery, indicating a probable association between periodontal disease and hematological dissemination from the oral cavity, leading to chorioamnionitis and its consequent effects on the fetus. A similar, but symptomatic case of a 31-year-old female at 21 weeks gestation, was reported from Canada [76]. This was the first reported case to reveal the practicability of performing 16S rRNA sequencing on formalin-fixed, paraffin-embedded placental tissues (FFPE) in microbiological identification of *F. nucleatum* and the first to establish the use of this molecular technique in the neonatal death investigation [76]. A study from Germany by Heusler et al. [65] proposed that low concentrations of *F. nucleatum* might promote the invasion of trophoblast cells and induce the secretion of mediators for pregnancy establishment. However, in contrast, unrestrained infections caused by *F. nucleatum* in early pregnancy might impact placental development. In a longitudinal study on pregnant Japanese women [77] real-time PCR with TaqMan probes and ELISA revealed that *F. nucleatum* levels in placenta samples were significantly higher in the threatened preterm labor (TPL) group compared to the healthy group before delivery. Additionally, the presence of *F. nucleatum* in placental tissues was found to be significantly higher in the TPL-Healthy delivery (HD) group compared to the healthy-HD group. The authors also affirmed the significant association of *F. nucleatum* in placental tissues with TPL, indicating it may be a potential risk factor. A review article from Qatar by Saadaoui et al. [49] threw the spotlight on the role of *F. nucleatum* in preterm birth, specifically in cases of clinical chorioamnionitis. The article also noted a temporal relationship between orogenital interaction with a male partner with periodontitis and the onset of clinical infection. Another review article from Italy [72] reported that meconium from preterm infants born to mothers with chorioamnionitis contained high levels of pathogenic bacteria, including *F. nucleatum*. Further, Payne et al. [66] from Australia demonstrated a significant enhancement of the microbial risk algorithm by the inclusion of *F. nucleatum* in the novel vaginal bacterial DNA test that was efficacious at the prediction of spontaneous preterm birth in a cohort of mostly white, low- to medium-risk Australian women during midgestational period. A study from China [78] -corroborated a significant positive correlation between the Apgar score (appearance, pulse, grimace, activity, and respiration) and the presence of *F. nucleatum* in the vagina. The Apgar score is a rapid method for assessing neonates immediately after birth or in response to resuscitation. A report by Walsh et al. [79] from the United States of America (USA) specified the role of *F. nucleatum* in triggering a more robust inflammatory response, leading to a higher incidence of preterm birth in African American women with a prevalence of *F. nucleatum* in their vaginal microbiome, compared to *L. crispatus*, which is prevalent in the vaginal microbiome of women of European ancestry. A case-control study from Brazil [67] conducted on 120 postpartum women, comprising 40 cases (gestational age < 37 weeks; preterm delivery) and 80 controls (gestational age ≥ 37 weeks; full-term delivery), revealed significantly higher proportions of *F. nucleatum* in the subgingival biofilm of cases compared to controls. Additionally, a 2022 cross-sectional case-control study in the USA [74] used umbilical cord blood specimens to evaluate the presence, abundance, and composition of bacteria through endpoint PCR of full-length 16S rRNA and the V4 amplicon sequence variants (ASVs). The study revealed *F. nucleatum* subsp. *animalis* as the most prevalent *F. nucleatum* strain detected in the umbilical cord blood microbiome in early preterm live birth cases.

The last five years have seen an increase in research on the link between *F. nucleatum* and spontaneous abortion/miscarriage, a pregnancy complication that may have physical and psychological effects on women [15,27]. In a matched case-control study conducted in Thailand [27], 85 women who experienced spontaneous abortion (<20 weeks of gestation) were compared to 85 controls of similar age, gestational age. The study found no significant differences in the levels of *F. nucleatum* in subgingival plaque between case and control groups. However, when subgroup analysis was conducted to compare levels of *F. nucleatum* between individuals with and without periodontitis in both case and control groups, with periodontitis had higher levels of *F. nucleatum* in both the spontaneous abortion and normal pregnancy groups.

*F. nucleatum* has been widely reported in various sources such as amniotic fluid, cord blood, fetal membranes, placental and fetal tissues of patients with stillbirth, fetal death, neonatal sepsis, and low birth weight [16,71,73,80]. It has also been found in vaginal samples of pregnant women at 28 weeks of pregnancy, and has been associated with early-onset neonatal sepsis [50]. In mice, *F. nucleatum* has been shown to induce stillbirths when given intravenously [25,47]. This is primarily caused by placental inflammation mediated by Toll-like receptor 4 (TLR4). In mice lacking TLR4 or treated with a TLR4 antagonist, *F. nucleatum* was found to colonize the placenta without eliciting an inflammatory response, resulting in a reduced fetal death rate [47]. In humans, *F. nucleatum* was isolated as a pure culture from the lung and stomach of a stillborn infant. An identical clone was also observed in the mother’s subgingival plaque, while no fusobacteria were detected in her vaginal and rectal flora [47].

Recent reports have also linked *F. nucleatum* to hypertension and preeclampsia [28,48,81,82,83,84,85]. Preeclampsia affects 5% to 7% of all pregnant women and is responsible for over 70,000 maternal deaths and 500,000 fetal deaths worldwide every year. It is the leading cause of maternal death, severe maternal morbidity, maternal intensive care admissions, caesarean section, and prematurity in the USA [86]. While higher levels of *F. nucleatum* have been associated with uncontrolled type 2 diabetes mellitus (T2DM), recent reports have not directly linked this bacterium to GDM. However, one recent review article has linked the genus *Fusobacterium* to GDM [48]. The above studies point towards the need for accurate species and strain-level identification of *Fusobacterium* in oral as well as vaginal microbiomes for predicting the risk of various APOs, which, in turn, might help to delineate appropriate therapeutic strategies. Moreover, clinical studies to reconnoiter the microbial profiles and the composition of multiple biofilms during the different trimesters of pregnancy should also be proposed.

*F. nucleatum*, detected in the neonatal microbiome, was found to be absent in the mother’s urogenital tract but present in their oral cavity. This suggests that the bacteria may have been transferred through the bloodstream due to its ability to adhere to vascular epithelium [87]. A review article from China [16] also highlighted that injecting saliva or subgingival plaque samples into mice can lead to placental infection with oral commensal species, including *F. nucleatum*, suggestingthat oral bacteria have the ability to travel to the feto–placental unit. Additionally, there is evidence of *F. nucleatum* detectionin amniotic fluid, placenta, and neonatal aspirates, matching those found in maternal oral samples [33,47]. These studies emphasize the importance of understanding the need to protect pregnant women from periodontal diseases. This further sheds light on the need to detect the environmental uniqueness of the placenta that permits it to harbor these potentially pathogenic oral commensals. These reports further corroborated the incidence of oral–uterine translocation via the bloostream [29,47,88].

Interestingly, recent reports have linked the abundance of *F. nucleatum* to various external factors like smoking, oral hygiene and diet [71,89,90,91,92]. Studies have shown that smokers have a higher abundance of *F. nucleatum* in their subgingival plaque samples compared to non-smokers [71,89]. Additionally, adherence to a Western diet, characterized by high fat and processed carbohydrate intake and low fiber consumption, has been linked to increased levels of *F. nucleatum* [90]. The consumption of inflammatory foods, such as refined grains, red and processed meats, and carbonated beverages, has also been associated with *F. nucleatum* infections [93]. In a review article from France, Martinon et al. [94] reported the significant bactericidal effects of a gel containing 1% curcumin (a bioactive substance of turmeric) on *F. nucleatum* in patients with periodontal diseases. A Polish study [37] stated that the use of zinc ions, commonly found in commercial anti-malodor mouthwashes, can inhibit *F. nucleatum*. A Columbian systematic review and meta-analysis conducted by Merchant et al. [95] indicated that treating maternal periodontitis patients infected with *F. nucleatum* using chlorhexidine mouthwash, scaling and root planing (SRP) reduces the risk of preterm birth and low birth weight babies. These reports highlight the importance of considering multiple external factors that impact a pregnant woman’s oral microbiota and potentially lead to APOs. This further emphasizes the need for a thorough understanding of the origin of the offending bacteria and their potential routes of invasion to the placenta and amniotic cavity, as well as the importance of maintaining good oral hygiene.

**Figure 2:**
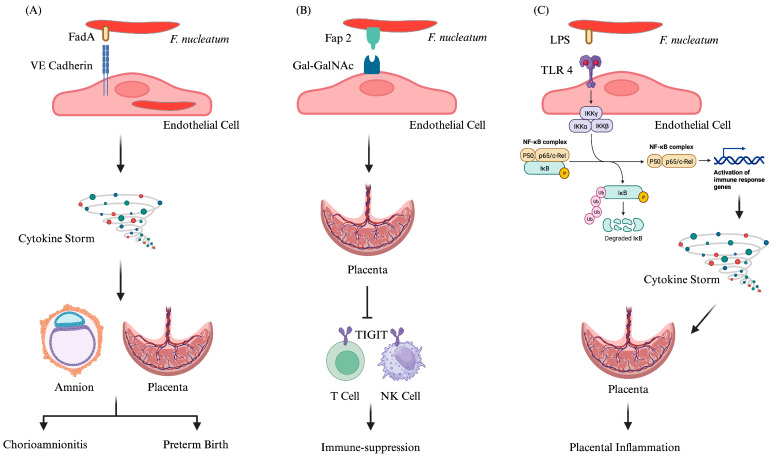
Role of *Fusobacterium nucleatum (F. nucleatum)* in adverse pregnancy outcomes (APO). (**A**) Interaction between *Fusobacterium* adhesion A (FadA), with vascular endothelial cadherin (VE-Cadherin) to internalize *F. nucleatum* in endothelial cells for bacterial dissemination, leading to increased inflammatory cytokines causing chorioamnionitis and preterm birth. (**B**) *Fusobacterium* apoptosis-inducing protein 2 (Fap2) binds with D-galactose-β (1–3)-N-acetyl-D-galactosamine (Gal-GalNAc) on endothelial cells to localize into placenta by suppressing TIGIT mediated activation of T cells and natural killer (NK) cells. (**C**) Lipopolysaccharide (LPS) interacts with TLR 4 on endothelial cells to activate the NF-κB pathway, leading to an inflammatory cytokine storm causing placental inflammation.

Several virulence factors and enzymes possessed by *F. nucleatum* are known to aid the bacterium in colonizing the fetal and placental membranes. However, Fusobacterium adhesion A (FadA), a unique adhesin of *F. nucleatum*, is best characterized and known to play a critical role in bacterial dissemination and colonization in the placenta. This is followed by spread to the fetal membranes, leading to acute inflammation of the placental and fetal membranes, causing chorioamnionitis and ultimately preterm birth [16,47,71] (Figure 2A). The active form of *Fusobacterium* adhesion A is an amyloid-like complex, termed FadAc, which comprises the intact pre-*Fusobacterium* adhesin A (129 amino acids) and the cleaved *Fusobacterium* adhesin A without the signal peptide. Under stress and disease conditions, *F. nucleatum* secretes amyloid-like *Fusobacterium* adhesin A, acting as a molecular switch to change the bacterium from a commensal to a pathogen [34,96]. The *fadA*-deletion mutant is significantly impaired in mediating placental colonization. The binding of FadA to vascular endothelial cadherin causes the loosening of the tight junction, allowing *F. nucleatum* and other oral bacteria to penetrate the endothelium. This may explain why *F. nucleatum* is often found in intrauterine infections, not only as a sole infectious agent but also concomitantly with other oral species [47].

Apart from FadA adhesin, *Fusobacterium* apoptosis-inducing protein 2 (Fap2) and the two Coaggregation regulator Response regulator and Sensor kinase– Arginine (R)- inhibitable adhesin D (CarRS- RadD) systems have been reported to be involved in *F. nucleatum* colonization of placenta [34]. Fap2, involved in interspecies coaggregation and cell adhesion, is a huge type V autotransporter with more than 3000 amino acids [34]. A recent study [29] involving human samples and a mouse pregnancy model has demonstrated that the fusobacterial outer membrane protein, Fap2, and host placenta displaying D-galactose-β (1–3)-N-acetyl-D-galactosamine (Gal-GalNAc) are involved in *fusobacterial* placenta localization and enrichment. Fascinatingly, Fap2-dependent *fusobacterial* attachment has been perceived not only in the placenta but also in the blood vessels leading to the placenta, signifying that enhanced Gal-GalNAc display is coordinated both in fetal and maternal tissues (Figure 2B). Interestingly, Fap2, mediating placental colonization by *fusobacteria*, is also found to activate the human (but not mouse) TIGIT killing-suppressing receptor expressed on T cells and natural killer (NK) cells (Figure 2B). Thus, galactose-sensitive adhesin, Fap2 of *F. nucleatum* contributes to its virulence for successful colonization in the placenta by evasion of host immune surveillance. However, a two-component signal transduction system, CarRS, consisting of the response regulator CarR and the sensor kinase CarS, regulates the expression of the outer membrane transporter adhesin RadD. Disruption or Interruption of CarR is reported to increase the fetal survival rate in mice, while the disruption of either CarS or RadD decreases their survival, unveiling a hypervirulence phenotype. It is still uncertain if the involvement of CarRS in placental infection is facilitated directly by RadDor through the regulation of other virulence factors, such as *Fusobacterium* adhesin A and/or Fap2 [34,68]. However, a recent study from USA [97] has reported the essential role of the multigene locus encoding a single, fused methionine sulfoxide reductase (MsrAB) and thioredoxin (Trx)- and cytochrome c (CcdA)-like proteins in attachment and colonization of *F. nucleatum* to the placental tissues, thereby expediting the initiation of infection and its spread to the amniotic fluid. This study explicitly adduces that the MsrAB system governed by the two-component system ModRS epitomizes a major oxidative stress defense pathway that keeps the bacteria away from oxidative damage in immune cells, thereby conferring virulence by enabling adhesion and invasion of target tissues.

Additionally, a study conducted by Garcia-So and colleagues from the USA [98] demonstrated that *F. nucleatum* triggers placental inflammation through maternal, TLR4-mediated signaling. The study revealed a spatiotemporal pattern of placental inflammatory response, with NF-κB activation (Figure 2C) first observed in maternal endothelial cells, followed by decidual cells surrounding the endothelium, and constant induction of inflammatory cytokines and chemokines. Moreover, the study highlighted the beneficial role of purified omega-3 fatty acids, specifically eicosapentaenoic acid (EPA) and docosahexaenoic acid (DHA), in suppressing inflammatory responses in endothelial cells mediated by both TLR2 and TLR4, thereby protecting against placental inflammation. Furthermore, the study showed that omega-3 fatty acids inhibit *F. nucleatum* proliferation in the placenta and increase fetal and neonatal survival. Therefore, Garcia-So et al. [98] not only elucidated the mechanism by which *F. nucleatum* causes intrauterine infection and triggers placental inflammation, leading to various APOs such as preterm birth, stillbirth, and neonatal sepsis but also provided a prophylactic measure to protect against such infections.

Furthermore, Park and colleagues [28] stated that receptor-interacting protein kinase 2 (Ripk2) might aid in the *F. nucleatum*-induced production of IL-6 by initiating NF-κB signaling in murine macrophages and human decidual stromal cells (hDSCs). Ripk2 was also reported to upregulate inducible nitric oxide synthase (iNOS) gene expression and NO production in macrophages. This promoted the production of CXCL8 and CCL2, which was reduced by Ripk2 inhibitors, SB203580 and PP2. These findings advocate the fact that APOs result from *F. nucleatum* infection due to the induction of aberrant production of cytokines and chemokines through nucleotide-binding oligomerization domain (NOD) 1/2-Ripk2-mediated signaling. This study also revealed that Ripk2 deficiency led to the reduced production of tumor necrosis factor α (TNF-α) in the absence of TLR4, thereby betokening the redundancy of Ripk2 in some immune responses in macrophages against *F. nucleatum*. This study [28] displayed for the first time the contribution of Ripk2 to cytokine or chemokine production in response to *F. nucleatum* in murine macrophages and hDSCs. It would be highly advantageous to regulate Ripk2 signaling to prevent APOs, especially in chorioamnionitis caused by bacterial infections and use it as a molecular drug target. However, further studies using animal models are required to expound the immunomodulatory role of Ripk2 in *F. nucleatum*-induced APOs and the beneficial role of Ripk2 inhibitors in the prevention of APOs. Moreover, the use of cutting-edge technologies is also warranted to fully unravel the complex regulatory networks of molecular and cellular events underlying the role of *F. nucleatum* in APOs. Table 1 summarizes the main findings from research articles and case reports linking the association between *F. nucleatum* and its potential adverse effects during pregnancy.

## 4. *F. nucleatum* in Gynecological Diseases

In the last 5 years, there have been several reports from various parts of the world indicating the involvement of *F. nucleatum* in various GDs like polycystic ovary syndrome (PCOS), endometriosis, and pelvic inflammatory disease (PID) like salpingitis and (BV) (please refer to Box 2) [15,30,31,32,75,99,100,101,102,103].

Box 2 Gynecological diseases 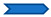 definition, histopathologies, etiologies, and symptoms.

**Polycystic ovary syndrome**

A complex endocrine and metabolic disorder classically characterized by anovulation, infertility, obesity, insulin resistance, and polycystic ovaries. It is also known as hyperandrogenic anovulation (HA) or Stein–Leventhal syndrome. It defines a typical condition where at least one ovary has an ovarian volume greater than 10 mL and at least one ovary has an estimated 12 small cysts, with diameters ranging from 2 to 9 mm [104]. However, due to advances in ultrasound technology, the initial Rotterdam criteria have been changed to >20 follicles in either ovary [105]. The most common contributing factors include obesity and insulin resistance. Fetal androgen exposure may also contribute to this condition [106]. According to the recently updated (2023) guidelines of the WHO, possible symptoms include heavy, long, intermittent, unpredictable, or absent periods, infertility, acne or oily skin, excessive hair on the face or body, male-pattern baldness or hair thinning, and weight gain, especially around the belly.

 

**Pelvic inflammatory disease**

Classically defined as an infection that originates in the cervical–vaginal region and spreads to the upper genital tract, resulting in a combination of symptoms such as acute salpingitis, perihepatitis, endometritis, oophoritis, pelvic peritonitis, and/or tubo-ovarian abscess [107]. The majority of PID cases are caused by sexually transmitted infections.

- **Salpingitis** is particularly defined as an infection and inflammation in the oviducts (fallopian tubes). These tubes are responsible for transporting oocytes and sperm, as well as facilitating fertilization and early embryonic development. The inflammation can be acute or chronic and can range in severity from mild to severe. It is typically caused by an infection that spreads from the lower tract to the upper genital tract [108]. It is also referred to as salpingitis isthmica nodosa (SIN) and is believed to be a part of the chronic pelvic inflammatory disease (PID) spectrum in some patients. Histopathologically, SIN is characterized by nodular thickening of the muscularis layer of the fallopian tube and the formation of inclusion cysts or diverticula due to overgrown epithelium. It is strongly associated with both infertility and ectopic pregnancies [109]. The current known causes of salpingitis include infection, cellular invasion, and congenital malformations [110]. Clinically, it is often manifested by edema, congestion of the fallopian tubes, and inflammation of the peritoneal structures [109].
- **Perihepatitis**, or Fitz–Hugh–Curtis syndrome, is a rare and chronic complication of PID that primarily affects premenopausal women. It is characterized by inflammation of the liver capsule and adhesion of the peritoneum, resulting in right upper quadrant pain [111]. The condition can be caused by various factors, including spontaneous ascending infection where microbes from the cervix or vagina travel to the endometrium, through the fallopian tubes, and into the peritoneal cavity; lymphatic spread, such as infection of the parametrium from an intrauterine device; and hematogenous spread, such as with tuberculosis. Common symptoms include acute pain and/or chronic tenderness in the right upper abdomen [112].
- **Endometritis** is an infectious inflammation of the endometrium, which is the innermost uterine layer. When the inflammation spreads into the muscular layer, the process is termed endomyometritis, and when it extends through to the parametrium, it is called endoparametritis. Histopathologically, acute endometritis is usually characterized by microabscesses and neutrophil invasion of the superficial endometrial epithelium, glandular lumens, and endometrial cavity. However, chronic endometritis is characterized by the infiltration of endometrial stromal plasmacytes (ESPCs), micropolyposis, edematous changes in the proliferative phase, and dissociated maturation between the stroma and epithelium. Additionally, B cells can accumulate in the endometrial stroma and glands. This condition is caused by the migration of normal bacterial flora from the cervix and vagina into the uterine cavity but can also be caused by bacteria from outside the genital tract. Symptoms of endometritis typically include irregular bleeding, pelvic discomfort, and leukorrhea [113].
- **Oophoritis** is a condition in which the ovaries become inflamed due to certain infections, potentially leading to impaired ovarian function. This inflammation can result in atrophic and fibrotic ovaries and, in rare cases, the replacement of ovarian stroma by foamy macrophages and histiocytes [114,115]. Various factors can contribute to the development of oophoritis, including sexual transmission, infection during pregnancy, and peripubertal infection, depending on the specific virus or pathogenic agent. Common clinical symptoms of oophoritis include anorexia, fever, suprapubic pain, menorrhagia, vaginal bleeding, adnexal tenderness, and/or a pelvic mass [114].
- **Peritonitis** is a medical condition characterized by inflammation of the peritoneum, which is the membrane that lines the abdominal cavity [116]. This inflammation can be caused by various factors, such as underlying health conditions or the presence of infectious agents. In some cases, it may also present as granulomas with central caseous necrosis, although this is rare. Some of the known causes of peritonitis include gastroduodenal perforations, intestinal volvulus, ruptured abscesses, traumatic bowel perforation, perforated peptic ulcers, tubo-ovarian abscesses, and amoebic colonic perforations [117]. The classic symptoms of peritonitis include severe abdominal pain, tenderness and rigidity, fever, chills, and altered mental status [118].
- **Tubo-ovarian abscess (TOA)** is a complex infectious mass that forms in the adnexa as a result of PID. It is often caused by bacteria from the lower genital tract that travel up to the fallopian tube, ovary, and potentially other nearby pelvic organs. Common risk factors include being of reproductive age, having an intrauterine device (IUD) inserted, having multiple sexual partners, and having a previous episode of PID. TOAs are typically polymicrobial and often contain a high proportion of anaerobic bacteria. Symptoms may include an adnexal mass, fever, elevated white blood cell count (WBC), lower abdominal or pelvic pain, and/or vaginal discharge [119].

**Endometriosis**

Defined as a chronic inflammatory hormone-dependent condition associated with pelvic pain and infertility. It occurs when uterine tissue grows outside of the uterus, leading to inflammation [120,121]. This can result in the formation of scar tissue, known as adhesions or fibrosis, in the pelvis and other parts of the body. The WHO has identified several types of endometriosis lesions, including superficial endometriosis on the pelvic peritoneum, cystic ovarian endometriosis (endometrioma) in the ovaries, and deep endometriosis in the recto-vaginal septum, bladder, and bowel. In rare cases, endometriosis may also be found outside of the pelvis. According to the WHO, the causes of endometriosis include retrograde menstruation, cellular metaplasia, and the spread of stem cells through the body via blood and lymphatic vessels. Symptoms, as described by the WHO, include severe pain during periods, sexual intercourse, bowel movements, and/or urination, as well as chronic pelvic pain, abdominal bloating, nausea, fatigue, and sometimes depression, anxiety, and infertility.

 

**Bacterial vaginosis**

Defined as a condition in which the vaginal microbiome presents an overgrowth of obligate and facultative anaerobes that disrupts the vaginal microbiome balance. It is a common and recurring infection among women of reproductive age and has been linked to adverse health outcomes and a decreased quality of life [122]. This condition is associated with significant negative healthcare outcomes, including an increased susceptibility to sexually transmitted infections, urogenital infections, pelvic inflammatory disease, and an increased risk of abnormal pregnancy [123]. Histopathologically, it is characterized by the presence of clue cells, which are epithelial cells of the cervix that are embedded with bacteria. It is typically caused by a decrease in the number of normal hydrogen peroxide-producing Lactobacilli, leading to an overgrowth of anaerobic bacteria [124]. Common clinical symptoms include a foul fishy odor and itching in the perineal region [125].

*F. nucleatum* has been frequently linked directly or indirectly to PCOS in several studies [15,31,99]. A systematic literature review from Macedonia [15] also found a high incidence of *F. nucleatum* in the saliva of PCOS patients. Similar facts have also been reported in a recently published chapter [126]. A nationwide cohort study conducted in Taiwan [92] did not directly link *F. nucleatum* to PCOS, however, it suggested the plausible association between chronic periodontitis in patients, often caused by periodontal pathogens such as *F. nucleatum*, and the development of the systemic antibody responses that might influence the pathogenesis of PCOS. Additionally, a case-control study from India [31] reported significantly elevated levels of *F. nucleatum* in PCOS patients with periodontitis and/or gingivitis. The authors of this study [31] concluded that hormonal imbalances in women with PCOS may impact their salivary levels of potential periodontal pathogens and systemic antibody responses, making them more susceptible to periodontal diseases such as gingival inflammation. These findings from different parts of the world support the idea that fluctuating sex hormones, combined with high levels of male hormones (androgens) in women with PCOS, may have a quantitative impact on the oral microflora, potentially leading to oral dysbiosis and contribute to gingival inflammation and periodontal health issues.

In the last five years, several studies have been published that have linked *F. nucleatum* to various PIDs [127,128,129,130,131]. One specific type of PID, salpingitis, has been found to be associated with *F. nucleatum* in sexually inactive patients [100]. A review article from India [132] also stated that *F. nucleatum* can cause different degrees of endometritis. In a case report from Canada, a 69-year-old woman with a 2-month history of postmenopausal bleeding and pelvic cramping was found to have *F. nucleatum* in her endometrial culture [101]. *F. nucleatum* has also been linked to endometritis in cows [133,134]. Reports from various parts of the world have stated the association of *F. nucleatum* with endometritis in either humans or animals, or both [135,136,137]. A 2019 case report [138] from the United Kingdom (UK), of a Pakistani woman with a 1-month history of lethargy, weight loss of 3 kg, and ascites, was reported to develop spontaneous bacterial peritonitis after 4 weeks of the initial ascitic sampling, with a positive culture of *F. nucleatum*. After a liver biopsy, she was diagnosed with “small hepatic vein” Budd–Chiari syndrome (BCS), which was found to be complicated by *F. nucleatum-*induced peritonitis. This case report further highlighted the possibility the patient might have developed peritonitis due to *F. nucleatum-*induced liver complications, owing to bowel content leakage traveling through the portal vein. Although no reports in the last 5 years have directly linked *F. nucleatum* with perihepatitis, oophoritis or TOA, other species of *fusobacterium* were reported to have associations with TOA [139].

**Figure 3:**
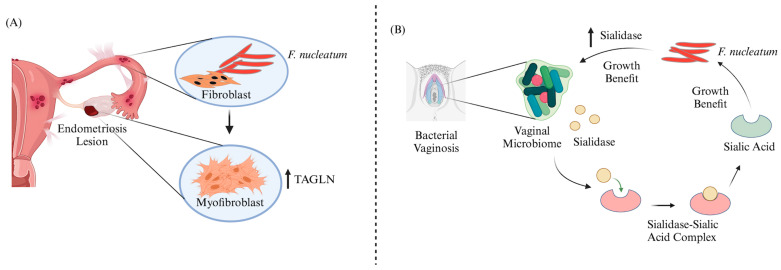
Role of *Fusobacterium nucleatum (F. nucleatum)* in gynecological diseases (GD). (**A**) *F. nucleatum* increases TAGLN expression in endometrial fibroblasts to convert them into endometriosis lesion-forming myofibroblast cells. (**B**) Schematic representation displaying the interdependent beneficial relationship between *F. nucleatum* and vaginal bacteria, ultimately leading to bacterial vaginosis.

A recent translational [30] study in Japan has demonstrated the pathogenic role of *F. nucleatum* in the development of endometriosis. The study found that 64% of women with endometriosis had *F. nucleatum* infiltration in the endometrium, compared to less than 10% of women without the disease. This is a benchmark publication that revealed that *F. nucleatum* infection of endometrial cells activates transforming growth factor–β (TGF-β) signaling, leading to the transition from quiescent fibroblasts to transgelin (TAGLN)-positive myofibroblasts, thereby conferring the bacterium with the ability to proliferate, adhere, and migrate in vitro. Additionally, Muraoka and colleagues [30] showed that *F. nucleatum* inoculation in a syngeneic mouse model of endometriosis caused a noticeable rise in TAGLN-positive myofibroblasts and increased the number and weight of endometriotic lesions (Figure 3A). This study also highlighted the potential of targeting *F. nucleatum* in the endometrium with antibiotic treatment as a therapeutic option for patients with endometriosis. This milestone work, which established a direct link between bacterial infection and endometriosis was featured in the news of ‘The Lancet Microbe’ [140] and the ‘Daily Briefing of Nature’ [141].

Several reports from various regions of the world have linked *F. nucleatum* to BV in the last five years [102,103,142]. Agarwal et al. [103] displayed that *F. nucleatum* foraging and growth on mammalian sialoglycans is facilitated by sialidase activity, which is a diagnostic feature of BV. This sialidase activity serves as a source of nutrients that would otherwise be inaccessible due to the lack of endogenous *F. nucleatum* sialidase. Additionally, their experiments on a mouse model revealed that *F. nucleatum* may also contribute to the community by enhancing sialidase activity, a biochemical feature of human dysbiosis (Figure 3B). This study also indicated that mutually beneficial relationships between vaginal bacteria can actually support the colonization of pathogens and may aid in maintaining features of dysbiosis. Thus, this study on BV is crucial, as its findings are discordant with the simplistic dogma that the absence of “healthy” lactobacilli is the sole mechanism that produces an accommodative environment for pathogens during vaginal dysbiosis. Furthermore, this study shed light on why women with BV are at a higher risk of vaginal colonization by pathogens such as *F. nucleatum.* Table 2 provides a summary of the key findings from research articles and case reports on the association between *F. nucleatum* and various GDs.

## 5. *F. nucleatum* in Gynecological Cancers

Recently, worldwide reports have stated the incidence of a high abundance of *F. nucleatum* in various types of GCs [breast cancer (BC), ovarian cancer (OC), endometrial cancer (EC), and cervical cancer (CC) (please refer to Box 3)]. Moreover, *F. nucleatum-*associated cancers are found to have poor prognostic value. A few of these reports also delineated the potential pathogenic role of this bacterium in the formation and progression of these cancers [14,24,142,143,144,145,146,147,148,149].

Box 3 Gynecological cancers 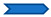 definition, types, histopathologies, etiologies, symptoms, and staging.

**Breast cancer**

Defined as the uncontrolled growth of epithelial cells originating in the ducts or breast lobules. Depending on its relationship to the basement membrane, it can be classified as either invasive or non-invasive. Non-invasive neoplasms are typically divided into two main types: lobular carcinoma in situ (LCIS) and ductal carcinoma in situ (DCIS). LCIS is identified by its adherence to the structure of a normal lobule, with enlarged and filled acini. On the other hand, DCIS is more varied in appearance and can be further categorized into four types: papillary, cribriform, solid, and comedo. Invasive ductal cancer typically presents as a cohesive mass and can appear as discrete abnormalities on mammograms. It is often palpable as a lump in the breast, typically smaller than lobular cancers. In contrast, invasive lobular cancer tends to spread through the breast in a single-file pattern, making it difficult to detect on mammography or physical examination until it has advanced. Tubular and mucinous tumors are typically low-grade (grade I) lesions, while medullary cancer is characterized by abnormal invasive cells with high-grade nuclear features, numerous mitoses, and a lack of an in situ component. BC develops due to DNA damage and genetic mutations, often as a result of exposure to estrogen. Inherited DNA defects or pro-cancerous genes, such as BRCA1 and BRCA2, can also increase the risk of developing BC. Therefore, a family history of OC or BC can increase an individual’s risk of developing BC. Other contributing factors may include advanced age, histologic abnormalities, early menarche, late childbirth, nulliparity, and late menopause [150]. According to WHO recommendations, the symptoms of BC can include a breast lump or thickening, often without pain, changes in the size, shape, or appearance of the breast, dimpling, redness, pitting, or other changes in the skin, changes in nipple appearance or the skin surrounding the nipple (areola), and abnormal or bloody fluid from the nipple. BC is classified using the TNM classification system, which groups patients into four stage groupings based on the size of the primary tumor (T), the status of regional lymph nodes (N), and the presence of distant metastasis (M) [150].

 

**Ovarian cancer**

Defined as a malignant neoplasm originating from the ovaries or fallopian tubes. It can be classified into different types, including epithelial, germ cell, stromal, and other types, such as mesothelial-mesenchymal, mixed cell, and secondary tumors [151]. The most common histological types of epithelial OC are serous, endometrioid, clear cell, and mucinous tumors. Less common subtypes include Brenner and seromucinous. Low-grade serous carcinoma (LGSOC) is a subtype of serous OC that is characterized by minimal nuclear atypia, rare mitosis, and fewer molecular abnormalities. On the other hand, high-grade serous carcinoma (HGSOC) is characterized by significant nuclear atypia, high mitotic activity (>12 per 10 high-power fields), and more molecular abnormalities as observed through cytogenetic analysis. LGSOCs are typically diagnosed at a younger age and have a better prognosis compared to HGSOCs, which tend to present at an older age. LGSOCs also have a higher frequency of KRAS and BRAF mutations, while HGSOCs have a higher frequency of p53 and BRCA 1 and 2 gene mutations and an absence of KRAS/BRAF mutations [152]. HGSOCs have a more aggressive clinical course and are genetically less stable [151]. Ovarian endometrioid carcinomas (OECs) are derived from endometriosis and are characterized by cystic areas that appear as soft masses with bloody fluid, as well as less common solid areas with extensive hemorrhage and necrosis. They also have microsatellite instability. OECs are typically diagnosed at an early stage, leading to a better prognosis [152]. Mucinous ovarian cancers (MOCs) are a unique subtype of OC with an unclear etiology, including whether they originate from the ovary or are the result of metastatic disease from other organs [153]. KRAS mutations are common in these tumors. Histopathological analysis may reveal the presence of glands with architectural and cytological features of adenocarcinoma, but they may lack stromal invasion [152]. Ovarian clear cell carcinoma (OCCC) is a rare subtype of epithelial OC with distinct molecular characteristics, specific biological and clinical behavior, poor prognosis, and high resistance to chemotherapy [154]. Histopathologically, OCCC shows cystic growth patterns, cellular clearing, and a characteristic hobnail growth pattern. Immunohistochemically, stage I and II tumors show overexpression of BAX, while metastatic lesions show higher expression of the anti-apoptotic protein BCL-2 compared to primary tumors. The most significant risk factor for OC is a positive family history of breast or ovarian cancer, and a personal history of BC also increases the risk. OC mainly affects postmenopausal women of advanced age. The symptoms of OC typically become apparent in the late stages (stage III or stage IV). This disease is often clinically manifested by a combination of symptoms, including abdominal fullness, bloating, nausea, abdominal distention, early satiety, fatigue, changes in bowel movements, urinary symptoms, back pain, dyspareunia, and weight loss. The staging of OC is determined by the 8th edition of the American Joint Committee of Cancer (AJCC) and the International Federation of Gynecology and Obstetrics (FIGO) staging system, which uses the TNM classification. This system has four stages, with each stage indicating increasing severity and decreasing chances of survival [152].

 

**Endometrial cancer**

Defined as a malignancy of the inner epithelial lining of the uterus [155]. EC is broadly classified into two types: Type I (association with unopposed estrogen stimulation, comprising low-grade cells that are more common and have a favorable prognosis) and Type II (not estrogen-driven, comprising high-grade cells that are less common and have an unfavorable prognosis). Type I ECs are mainly composed of grade I or grade II endometrioid adenocarcinomas, while Type II ECs comprise grade III endometrioid adenocarcinomas, serous, clear cell, undifferentiated, and carcinosarcomas [155]. Type I, low-grade endometrioid carcinomas (LGECs) are usually confined to the uterus during diagnosis and have a comparatively favorable prognosis compared to high-grade serous endometrial carcinomas (HGSECs) and other type II endometrial carcinomas, which have significantly poorer prognoses and are often disseminated at the time of diagnosis. The majority of endometrial LGECs show microsatellite instability (MSI) and carry PTEN mutations, while HGSECs commonly exhibit nuclear pleomorphism and nearly ubiquitous TP53 mutations. ECs with high-grade solid, endometrioid, and/or transitional cell-like (SET) morphologies resemble pure HGSC. Many HGSC-SET carcinomas also display evidence of TP53 mutations [156]. Endometrial clear cell carcinoma (ECCC) is a rare and aggressive type II endometrial carcinoma that is more common in older women and patients with advanced-stage disease [157]. It is typically characterized by a combination of papillary, tubulocystic, and/or solid architectural patterns, with cuboidal or polygonal cells containing nuclei with varying degrees of pleomorphism (although overt pleomorphism is usually absent). Hobnail tumor cells and cytoplasmic clearing are often present but are not necessary for diagnosis [158]. Endometrial undifferentiated carcinomas (EUCs) are composed of sheets of monotonous, typically dyscohesive cells that may have a rhabdoid appearance. They often have a limited expression of cytokeratins and are generally negative for epithelial membrane antigen, PAX8, and hormone receptors. They also lack membranous E-cadherin and typically demonstrate loss of expression of DNA mismatch repair proteins and SWI-SNF chromatin remodeling proteins [158]. Endometrial carcinosarcoma is a rare and immensely aggressive disease characterized by a biphasic growth of malignant epithelial (carcinomatous) and mesenchymal (sarcomatous) components [159]. Carcinosarcomas are classified into homologous and heterologous types, depending on whether the mesenchymal component displays differentiation that is intrinsic (endometrial stromal sarcoma or leiomyosarcoma) or extrinsic (chondrosarcoma, rhabdomyosarcoma, etc.) to the uterus [158]. Possible etiologic factors for EC may include exposure to endogenous or exogenous estrogen unopposed by progesterone or progestins, insulin resistance, and hyperandrogenemia [156]. Abnormal vaginal and postmenopausal bleeding are the most commonly reported symptoms, often accompanied by abdominal complaints, vaginal discharge, uterine prolapse, and urinary incontinence [160]. The extent of EC is surgically pathologically “staged” according to the International Federation of Gynecology and Obstetrics (FIGO) criteria published in 2009 [156]. It has four stages, with increasing severity.

 

**Cervical cancer**

Defined as a malignant tumor of the cervix that can be divided into two histological types, adenocarcinoma (AC) and squamous cell carcinoma (SCC). Human papillomavirus (HPV) is the primary cause of CCs, accounting for more than 75 percent of cases, with high-risk HPV 16 and 18 being the most common types [161]. The majority of squamous cell carcinomas (SCCs) are also HPV-positive. Histopathologically, invasive SCCs of the cervix present as a network of anastomosing bands or single cells with intervening inflammatory or desmoplastic stroma. The cervical stroma in the tumor is typically infiltrated by plasma cells and lymphocytes, with rare instances of an eosinophilic response or foreign body type giant cell reaction [162]. According to the World Health Organization (WHO), HPV-independent SCCs are defined as squamous tumors with stromal or exophytic invasion and negative P16 immunohistochemistry (IHC). These types of SCCs have a higher rate of node metastases and a poorer prognosis [161]. HPV-associated adenocarcinomas can be identified by the presence of conspicuous apical mitoses and karyorrhexis at low power magnification. These adenocarcinomas are classified into two histological types: usual and mucinous. The usual type is the most common and includes glands with smooth luminal surfaces and pseudostratified columnar epithelial cells with enlarged stretched-out, and hyperchromatic nuclei. The mucinous type is further subtyped into mucinous, not otherwise specified (NOS) adenocarcinoma, intestinal adenocarcinoma, signet-ring cell adenocarcinoma, and invasive stratified mucin-producing carcinoma [163]. HPV-independent adenocarcinomas are negative or patchy for P16. These types of adenocarcinomas are typically diagnosed at a later stage, with extrauterine spread and a higher prevalence of destructive invasion [162]. Contributing factors for HPV-associated CC include early age at first intercourse, multiple sexual partners, smoking, herpes simplex virus (HSV), human immunodeficiency virus (HIV), co-infection with other genital infections, and oral contraceptive use [161]. According to the WHO guidelines (2022), symptoms of early-stage CC include irregular bleeding or spotting between periods in women of reproductive age, postmenopausal bleeding or spotting, bleeding after sexual intercourse, and increased vaginal discharge, sometimes with a foul odor. As the cancer progresses, more severe symptoms may appear, such as persistent back, leg, or pelvic pain, weight loss, fatigue, loss of appetite, foul-smelling discharge, and vaginal discomfort, and swelling of one or both lower extremities. The degree of CC is pathologically divided into four stages using the FIGO staging system.

**Figure 4:**
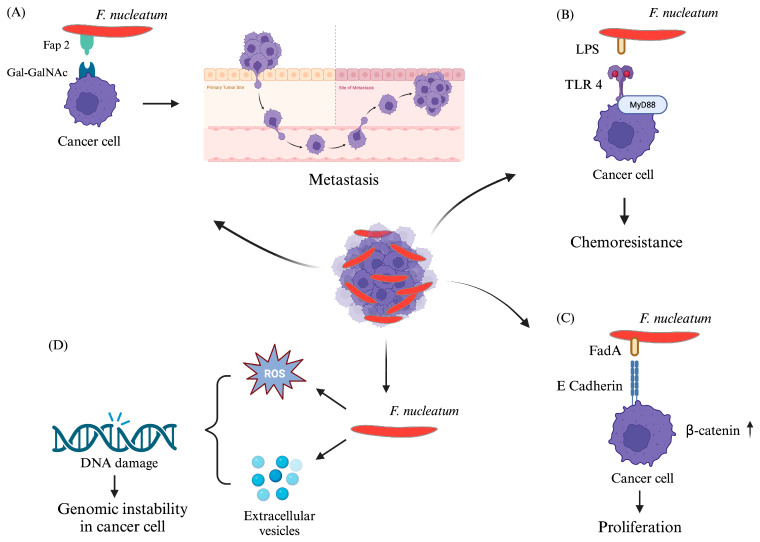
Role of *Fusobacterium nucleatum* (*F. nucleatum*) in gynecological cancers (GC). (**A**) *Fusobacterium* apoptosis-inducing protein 2 (Fap2) binds with D-galactose-β (1–3)-N-acetyl-D-galactosamine (Gal-GalNAc) on cancer cells to induce metastasis from primary tumor sites to other organs. (**B**) Lipopolysaccharide (LPS) on *F. nucleatum* activated TLR4/MyD88 pathway to induce chemoresistance in cancer cells. (**C**) Interaction between *Fusobacterium* adhesion A (FadA), with epithelial cadherin (E Cadherin) on cancer cells to increase b-catenin expression and induce cancer cell proliferation. (**D**) Reactive oxygen species (ROS) and extracellular vesicles secreted from *F. nucleatum* induce DNA damage in cancer cells to acquire further mutation and genomic instability.

In recent years, a plethora of articles have been published, which have given multifarious views regarding the direct and indirect role of *F. nucleatum* in BC development, progression and its prognostic outcomes [16,24,33,42,80,134,143,148,157,164,165,166,167,168,169,170,171,172,173,174,175,176]. A few of the current reports [14,24,26,167,177,178,179,180,181] have stated that BC tissues are found to be enriched with *F. nucleatum* and the bacterial load progressively increases with the tumor size and the advanced stages of cancer. These reports have suggested that *F. nucleatum* may contribute to poorer clinical outcomes and shorter survival. In a study conducted by Bernhard et al. [143] in Brazil, 44 women with BCs were evaluated for the microbial abundances of over 40 bacterial species within the subgingival plaque samples (n = 144). *F. nucleatum* was found to have one of the highest mean counts of infectious pathogens in the samples. This study indicated a strong association between oral *F. nucleatum-*induced chronic inflammation and BC, suggesting it as one of the contributing factors for female BCs. A cohort study by Nejman et al. [182] in Israel reported that *F. nucleatum*, earlier found to be enriched in colorectal tumors, is also predominant in the breast tumor samples. This study highlighted that the organs such as the breast, which was previously considered sterile, has the potential to harbor microbes. Further, a recent review and meta-analysis based on women between the ages of 18 and 96 years from the Netherlands, has directly pointed out the significant relationship between oral *F. nucleatum* species and their critical role in female-specific BC pathogenesis, thereby accentuating their biomarker potentiality [171]. Furthermore, an earlier review article from the USA [183] used a random forest classifier and identified *F. nucleatum* as one of the 14 potential microbial markers for postmenopausal women with BC. Contradictorily, a review article by Chadha et al. [164] from India specified that urine and fecal microbiome analyses of postmenopausal BC patients exhibited reduced counts of *F. nucleatum*. Another study from India [184] did not find an enrichment of *F. nucleatum* in BC transcriptome samples. Nonetheless, it is worth noting that *F. nucleatum* strain ATCC 23726 has been shown to specifically colonize mouse BC tissues and promote tumor growth and metastasis [14,185]. In a groundbreaking publication by Parhi and colleagues [14], it was documented that oral *F. nucleatum* species can translocate and colonize the lactiferous ducts in breast tissues through a hematogenous route. This colonization relies on neoplastic tissues expressing D-galactose–β (1–3)-N-acetyl-D-galactosamine (Gal-GalNAc), which are extensively displayed on BC cells. Their scholarly work using experimentations executed on murine models exhibited that the high Gal-GalNAc level in BC tissues, acting as an oncoantigen, plays a critical role by serving as a ligand to Fap2 (a surface lectin from *F. nucleatum*) in the specificity of tumor colonization by *F. nucleatum*. This, in turn, promotes mammary tumor growth and its metastatic progression (Figure 4A). They further advocated that this effect may be mediated by the prevention of the accumulation of tumor-infiltrating T cells in the tumor microenvironment (TME) and/or increased expression of matrix metalloproteinase-9 (MMP-9). Additionally, their work also revealed that antibiotic therapy using metronidazole can counteract the metastatic progression of mammary tumors, thereby suppressing BC aggravation. This work further highlighted the fact that targeting *F. nucleatum*, specifically Fap 2, might be advantageous in the treatment of BCs. In this study, the researchers utilized a method to visualize bacteria in tissue slices from an in vivo model of BC. This method has been recently published [186], and provides direct visualization of *fusobacterial* colonization in BC tissue using multiphoton microscopy. The establishment and validation of this protocol by Parhi and colleagues has gained significant histopathological importance in recent times, as it enables direct visualization without causing any damage to the tissues, allowing for the identification of all structures. Furthermore, a review by Van der Merwe and colleagues [187] from South Africa that elucidated the possible mechanisms by which *F. nucleatum* might promote BC progression, also speculated that this particular bacterium may promote BC progression by activating the TLR4/MyD88 pathway through its immunomodulatory effects, as indicated in the literature for colorectal cancer. However, this hypothesis proposed by Van der Merwe and colleagues requires further investigation and validation. If the onco-immunological implications of *F. nucleatum* are confirmed through experimental research, it could provide valuable insights into the microbial and immune-therapeutic aspects of this disease, potentially leading to advanced treatment options. This review further professed thatnovel methods of impeding the binding of *F. nucleatum* to tumors such as Gal/GalNAc antagonists or Fap2 antibodies should be considered. Withal, a recent study from China [179] using in vivo experimentation techniques, has validated that the *F. nucleatum-*derived small extracellular vesicles (*F. nucleatum*-EVs) can significantly augment the cell viability, proliferation, migration, and invasion of BC cells, thus inducing a promotive function on mammary tumor growth and metastasis. They also found that knocking down TLR4 in BC cells efficaciously counteracted the effects of *F. nucleatum*-derived EVs. This indicated the TLR4 activation-dependent contributive role of *F. nucleatum*-derived EVs in BC tumor growth and metastasis. Furthermore, exosomes, which are small extracellular vesicles (EVs), play a crucial role in mediating cellular communication by delivering various bioactive molecules, such as oncogenes, oncomiRs, proteins, and even pharmacological compounds. These molecules can be transferred to target cells, altering their transcriptome and influencing tumor-related signaling pathways. While numerous studies have investigated the involvement of exosomes in BC biology, including therapeutic resistance and the surrounding microenvironment [188], the role of exosomes derived from *F. nucleatum* in BC progression remains largely unexplored. However, a study by Guo et al. [189] from China demonstrated that exosomal miR-1246/92b-3p/27a-3p derived from *F. nucleatum*-infected CRC cells promotes metastasis in uninfected cells, contributing to CRC progression. Additionally, a recent study from China [190] has identified that *F. nucleatum*-infected gastric cancer (GC) cells produce exosomes that increase the expression of the long non-coding RNA (lncRNA) HOXA transcript at the distal tip (HOTTIP), promoting GC invasion through the miR-885-3p/EphB2/PI3K/AKT axis. Therefore, further research is needed to fully understand the role of *F. nucleatum*-derived EVs in BC and their associated pro-inflammatory and inflammatory responses, which could potentially lead to the development of novel therapeutic agents. A 2023 review article [178] examined the molecular consequences of the *F. nucleatum* within the TME, likely indicating the probable actionable pathways modulated by this anaerobic bacterium that may have significance in BC patients. Little and her colleagues have put forward a question to the new age oncologists and cancer researchers concerning whether *F. nucleatum* is capable of modulating the local TME, promoting an inflammatory state and further interacting with and influencing infiltrating immune cells in the case of BC as suggestive of CRC in the literature. They also recommended the use of advanced in vitro models such as organoids to replicate the hypoxic environment of the tumors and study the impact on the survival and growth of the anaerobic bacterium *F. nucleatum*. A three-dimensional (3D) tumor spheroid model was successfully used to examine the effects of co-culturing viable *F. nucleatum* with human epithelial colon cancer cells, including gene expression, metabolomics, and morphology [191]. Little et al. [178] also highlighted the need for the development of cost-effective assays to detect and quantify *F. nucleatum* in BC patients. Their review work emphasized that it may be of great interest to immunobiologists and cancer researchers to unravel the immunogenetics concerning the potential interaction between *F. nucleatum* and immune checkpoint inhibitors (ICI) in the breast. Another review article [192] also highlighted that formate-producing *F. nucleatum* stimulates the aryl hydrocarbon receptor (AhR) signaling pathway, thereby promoting cell migration and eliciting cancer stem cell (CSC) traits, high metastatic activity and active Wnt signaling. This pro-malignant effect of AhR ligands was seen in the BC cell line MCF-7. Thus, these works provided valuable insights into the immunomodulatory role of the *F. nucleatum* in BC development, progression and its effect on treatment effectiveness in patients.

In recent years, several reports have been published linking *F. nucleatum* to OCs [144,146,147,193]. One such case series, presented by Almohaya et al. [144] from Saudi Arabia, highlighted the incidence of *F. nucleatum*-associated bacteremia in a 72-year-old female patient with metastatic OC. The patient had a high white blood cell count (28.4 × 10^9^) and c-reactive protein level (124 mg/L), anddespite receiving 14 days of antibiotic treatment (meropenem and moxifloxacin), she passed away within 30 days. Another study in 2022 from Korea [146] reported the incidence of *F. nucleatum-*associated bacteremia in an OC patient. However, the bacterial isolates were found to be susceptible to all the 10 antimicrobials tested that belonged to the groups: penicillin, cephalosporins, carbapenems, beta-lactamase inhibitors, macrolides, fluoroquinolones, chloramphenicol, and metronidazole. Additionally, a study from China [193] summarized the clinical characteristics of patients with *F. nucleatum* infection. The study reported the bacterium’s association with a female patient aged below 70 years suffering from OC who died within 2 days. A recent study from the USA [147] has utilized the differential abundant analysis to demonstrate the enrichment of *F. nucleatum*, in patients with other OC histologies, in comparison to the serous OC patients within the whole OC cohort. These studies not only depicted a possible relationship between *F. nucleatum* infection and OC, but also provided clinically appropriate data for the implementation of empirical therapies against *F. nucleatum*. CXC motif chemokine ligand 16 (CXCL16), a chemotactic cytokine belonging to the α-chemokine subfamily, and its receptor CXC motif chemokine receptor 6 (CXCR6) have been shown to play significant roles in the progression of various cancers (invasion and migration), including OCs [189,194]. Further, in a study by Guo et al. from China [189], *F. nucleatum* was found to stimulate the production of CXCL16 in CRC patients. The observations from these studies suggested that *F. nucleatum* can stimulate the production of CXCL16, which can further activate CXCR6 and potentially aid in the implantation of OC cells and the formation of peritoneal metastasis. However, this plausible chemokine-related *F. nucleatum-*induced initiation and formation of OCs need experimental validation in future studies which, in turn, is expected to open avenues to target OCs by controlling immune cell trafficking. Moreover, CSCs have an imperative role in ovarian tumor initiation, invasion, metastasis, local recurrence following curative resection and therapeutic resistance. The process of epithelial–mesenchymal transition (EMT) is also considered a vital step in OC proliferation and CSC metastases [195,196]. EMT is characterized by the repression of E-cadherin (an important component of adherens junctions), occludins, claudins, Epcam, α6β4 integrin, and different cytokeratins (important for stabilization of desmosomes) and up-regulation of vimentin, fibronectin, neural cadherin (N-cadherin), β1 and β3 integrins, and matrix MMPs [197]. The regulatory crosstalk between CSC and EMT is known to increase cancer cell mesenchymal characteristics on the CSCs and help to promote OC cells to gain stemness [196]. Studies have also reported *F. nucleatum* can induce CSC characteristics by activating IL-6/STAT3 and eliciting EMT-resembling activation [196,198]. Another report from China [199] showed that *F. nucleatum* significantly upregulated the expression of lncRNA Keratin7-antisense (KRT7-AS) and Keratin7 (KRT7) in CRC cells. However, it is currently unclear whether KRT7-AS is directly involved in the initiation and development of OC, but earlier, it was reported that KRT7 regulated EMT in OC via the TGF-β/Smad2/3 pathway [200]. It is also known to regulate cell-matrix adhesion through integrin-β1-focal adhesion kinase signaling, signifying numerous potential links between KRT7-AS and OC [200,201]. In OC, silencing of the TLR4 gene expression is known to cause a reduction in the expression of MMP2 and MMP9 and decrease levels of mesenchymal markers in lipopolysaccharide (LPS)-treated OC cells [202]. TLR4/MyD88 signaling is also found to be associated with chemoresistance to paclitaxel in OC (Figure 4B) [203]. On top of that, *F. nucleatum* has been reported to be targeted by TLR4-mediated innate immune signaling and alters the chemotherapeutic response in CRC patients [202,204]. While these studies could not directly identify the pathways involved in *F. nucleatum*-induced OCs, they shed light on the probable ones, thereby accentuating the need for further exploration. Additionally, a study from the USA [142] highlighted an earlier observation that lanthionine (highly accumulated during *F. nucleatum* infection) biosynthesis, which generates hydrogen sulfide (H_2_S), is associated with advanced-stage OC in a mouse model. These studies strongly implied the probable mechanistic association between *F. nucleatum* and OCs and opened the way for the discovery of new therapeutics by targeting biological pathways.

Although there have not been many recent studies directly linking *F. nucleatum* to ECs, two recent reports published in 2022 [205] and 2023 [149] from Florida and Poland, respectively, have strongly professed a high abundance of this bacterium in vaginal and/or cervical samples collected from women suffering from EC. The exact mechanism by which *F. nucleatum* contributes to the development and progression of ECs, either directly or indirectly, is still unknown. However, this review proposes a few potential causes that should be further investigated in future studies. In patients with CRC, the presence of *F. nucleatum* is known to activate the Wnt/β-catenin signaling pathway [205]. This pathway is crucial for normal cellular proliferation during the menstrual cycle, but when it is dysregulated in the endometrium, it can lead to endometrial hyperplasia and potentially EC. Thus, the observations mentioned above indicate the potential carcinogenic role of *F. nucleatum* in the development of ECs through oncogenic activation or induction of aberrant Wnt/β-catenin signaling (Figure 4C). Additionally, type 1 ECs are often known to be associated with some unique molecular alterations, also termed microsatellite instability (MSI), which is the result of the defects in DNA mismatch repair proteins. This is due to the accumulation of mutation loads in cancer-related genes and the generation of neoantigens, which stimulate the anti-tumor immune response of the host [206,207,208,209]. Moreover, *F. nucleatum* infection has also been linked to MSI in CRC and head and neck cancer patients due to impaired DNA mismatch repair (MMR) pathways [210,211,212]. Thus, mechanistically, the capacity of *F. nucleatum* to create genotoxic oxidant species by causing epigenetic changes and inducing inflammation-associated microsatellite instability via impairment of MMR to promote DNA damage and cell proliferation, may possibly underlie the pathobiont’s associations with ECs (Figure 4D). Further exploration of these target pathways concerning *F. nucleatum* and EC may provide valuable therapeutic insights into ECs. Furthermore, studies have shown that abnormal expression of lncRNAs is involved in the development of ECs by affecting pathways related to the cell cycle, DNA replication, and mismatch repair [213,214]. Additionally, the process of EMT in ECs has been linked to the expression of transcription factors such as Snail, Slug, Twist2, Zeb1, and Zeb2 [215,216]. While no direct link has been established between *F. nucleatum* and the expression of lncRNAs in promoting EC, a recent study from China [217] has demonstrated that *F. nucleatum* can promote CRC metastasis by upregulating the expression of lncRNA endogenous retroviral-associated adenocarcinoma RNA (EVADR). This lncRNA acts as a scaffold for Y-box binding protein 1 (YBX1) which, in turn, enhances the translation of EMT-related factors such as Snail, Slug, and Zeb1. Therefore, Lu and colleagues have highlighted the crucial role of *F. nucleatum* in regulating lncRNAs and EMT-related factors. This not only provides new insights into the molecular mechanisms underlying CRC metastasis but also suggests that exploring the dysregulated expression of lncRNAs induced by *F. nucleatum* in EC cells may lead to new therapeutic targets for treating patients with EC.

The association of *F. nucleatum* with CC has been implicated in a few studies published in the last 5 years from various parts of the world [16,142,145,172]. This pathobiont is mostly found in patients suffering from HPV-related CC, cervical intraepithelial neoplasia, and invasive cervical carcinoma [142]. In a 2020 study conducted in China on 112 patients with squamous carcinoma of the cervix, high levels of *F. nucleatum* were found, particularly in cases of recurrent lesions [145]. Apart from this, Huang and colleagues made several novel observations. They found that patients with high burdens of intratumorally infiltrated *F. nucleatum* displayed poor rates of both overall survival and progression-free survival (PFS). Thus, this anaerobic bacterium can serve as a potential CC diagnostic and prognostic biomarker. Interestingly, the levels of *F. nucleatum* were positively correlated with tumor differentiation. These observations by Huang et al. [145] might help to improve or change therapeutic strategies and provide a better prognostic outcome for afflicted patients. Furthermore, the study showed that CC cells from patients with high levels of intratumorally infiltrated *F. nucleatum* exhibited characteristics of CSCs. Their scholarly work also implied that induction of CSC characteristics by *F. nucleatum* might be through the activation of certain specific transcription factors, such as NANOG, Octamer-binding transcription factor 4 (OCT4), SRY-Box Transcription Factor 2 (SOX2) and associated signaling pathway, such as WNT/β-catenin and insulin-like growth factor 1 (IGF-1) receptor pathway. Hence, increased *F. nucleatum* burden might further activate many other sequences of metastasis, such as C-X-C Motif Chemokine Receptor 4 (CXCR4), epithelial cellular adhesion molecule (Ep-CAM), Slug, Snail1 and Zinc Finger E-Box Binding Homeobox (Zeb1/2). Therefore, this study by Huang et al. [145] not only delineated a cogent role of *F. nucleatum* in the onset of CC and its consequent development and progression but also betokened a plausible role of the *F. nucleatum* in the dynamics underlying CCs. Thus, Huang and his colleagues provided appropriate rationale and merit to further investigate their findings in a larger cohort and explore novel mechanisms concerning *F. nucleatum-*associated CCs to aid inthe development of better treatment options for CC patients. Table 3 outlines the specific findings from research articles and case reports on the link between *F. nucleatum* and various GCs.

## 6. Conclusions, Future Challenges, and Perspectives

*F. nucleatum*, an opportunistic pathogen, and also one of the most variegated bacterial species, has recently been found to play a significant role in various infectious and systemic diseases, including tumorigenesis, particularly in colorectal and breast carcinomas. This has accrued considerable attention over the past decade; however, so far, the growing pieces of evidence remain full of correlative observations and associations that immensely outnumber the field’s mechanistic studies. To the best of our knowledge, this literature review is the first one to identify a knowledge gap relating to the mechanistic role of *F. nucleatum* in various aspects of gynecology, particularly OCs and ECs. Addressing this gap in future studies could provide valuable insights into the basic biology of *F. nucleatum*-associated GC initiation and progression and shed light on its potential pathogenic, mechanistic and contributory roles in these diseases. Thus, this review highlights the need for further mechanism-driven research, which may garner the attention of clinicians and researchers. Further, this is the first review of its kind that successfully provides a well-defined overview of the present developments of *F. nucleatum* concerning its epidemiological evidence and mechanistic linkage in almost all notable GDs, including cancers. Moreover, here, we not only summarize the detrimental effects of *F. nucleatum* on women’s health but also discuss the available treatment options and possible therapeutic strategies to combat *F. nucleatum* infections causing or contributing to various APOs and GDs, including cancers. Numerous recent findings discussed in this review have shed light on the interplay between *F. nucleatum*; APOs, *F. nucleatum*; GDs and *F. nucleatum*; and GCs, some of which may break the ground for promising novel therapies, especially for GCs.

Nevertheless, there is still much to be discovered in order for this field to progress. Several epidemiological studies reviewed here only showed the correlation between *F. nucleatum* and various APOs and GDs, inclusive of cancers. However, the explicit causation and mechanisms of pathogenesis of *F. nucleatum* in relation to diseases impacting women’s health, as well as its sensitivity to immunomodulation and immune escape, have yet to be uncovered. A few conclusions obtained in various studies were contentious. Therefore, in order to identify targeted therapies for *F. nucleatum*-associated APOs and other GDs, including cancers, researchers must delve deeper into the basic biology of *F. nucleatum*, not only in the field of gynecology or gynecological oncology, but also in its natural habitat and other disease-associated conditions and locations. It is also important for scholars to identify the pathways or mechanisms through which *F. nucleatum* interacts with its host. Immune mechanisms involved in this phenomenon deserve further exploration. Preclinical models, especially organoids and humanized gnotobiotic mouse models that mimic human tissue-specific microenvironments have been recently used to study human cancer genetics and the human microbiome. However, their use to study the effects of *F. nucleatum* in various GDs including GCs, is yet to be executed.

Currently, advancements in detection methods for microbial entities and microbe-derived small molecules have greatly improved our ability to accurately identify and quantify *F. nucleatum* strains and levels in the mouth and gut. This is particularly crucial for pregnant women and immunocompromised individuals. By understanding the bacterial load and the molecular mechanisms involved in the transformation of this oral commensal into a pathobiont, which can impact women’s health, researchers and clinicians can not only develop effective diagnostic and therapeutic strategies but can also educate women on preventive measures. The potential for using *F. nucleatum* as a disease-predictive biomarker in various GDs, including carcinomas, is promising. However, there is still much to be discovered and explored regarding the interactions between *F. nucleatum* and the host, which ultimately determine a female patient’s response to a specific treatment regimen and, potentially, their long-term impact on women’s health.

## Figures and Tables

**Table 1:** Summary of research articles and case reports linking *F. nucleatum* to APO.

SL. No.	Article Type	Country of Report	Year of Publication	Specific Findings/Main Highlights	References
1	Research article	Thailand	2019	Association between *F.* nucleatum and spontaneous abortions in individuals with periodontitis.	Chanomethaporn et al. [27]
2	Case report	Canada	2019	Association between *F. nucleatum* and a symptomatic case of acute chorioamnionitis and PROM; first report to use a specific molecular technique in neonatal death investigation.	Chan et al. [76]
3	Research article	USA	2019	Omega-3 fatty acid is a promising prophylactic therapy to protect against intrauterine infections, as it has been shown to suppress *F. nucleatum*-induced placental inflammation, both in pregnant mice and in vitro, using human umbilical cord endothelial cells.	Garcia-So et al. [98]
4	Research article	Japan	2020	A significant association between *F. nucleatum* and TPL in placental tissues.	Ye et al. [77]
5	Research article	USA	2020	A greater prevalence of *F. nucleatum* in the vaginal microbiome of African American women is capable of initiating the inflammatory response that might result in preterm birth.	Walsh et al. [79]
6	Research article	Korea	2021	*F. nucleatum* infection induces innate inflammatory responses in macrophages hDSCs and results in APOs by induction of aberrant production of cytokines and chemokines through NOD1/NOD2-Ripk2-mediated signaling, suggesting Ripk2 signaling as a potential preventive and therapeutic target against APOs.	Park et al. [28]
7	Research article	Australia	2021	Significant improvement in risk prediction for spontaneous preterm birth by inclusion of *F. nucleatum* in test algorithm.	Payne et al. [66]
8	Research article	USA	2021	The adhesin RadD, a major virulence factor of *F. nucleatum*, not only mediates polymicrobial interaction (or coaggregation) but is also critical in a mouse model of preterm birth.	Wu et al. [68]
9	Research article	Israel	2022	Galactose-sensitive adhesin, Fap2 of *F. nucleatum* contributes to its virulence for successful colonization in the placenta by selectively binding to Gal-GalNAc, also called T antigen as this antigen is over-displayed during fetal development.	Parhi et al. [29]
10	Research article	USA	2022	*F. nucleatum* has been detected as the most prevalent species in cord blood in early preterm live birth cases.	Vander Haar et al. [74]
11	Research article	China	2022	*F. nucleatum* in the vagina can serve as a potential biomarker for APO.	Sun et al. [78]
12	Research article	USA	2022	*F. nucleatum* possesses a multigene locus encoding a fused MsrAB and the associated factors Trx/CcdA that help the bacteria colonize the placenta and spread to the amniotic fluid to induce preterm birth in a murine model.	Scheible et al. [97]
13	Research article	Brazil	2023	A significantly higher proportion of *F. nucleatum* in the subgingival biofilm of the women with gestational age < 37 weeks compared to those with gestational age ≥ 37 weeks.	Lima et al. [67]
14	Case report	Italy	2023	A minor dental procedure may contribute to the development of *F. nucleatum-*associated chorioamnionitis and PPROMwithout any prior symptoms in the mother.	Bonasoni et al. [75]

Abbreviations: APO—adverse pregnancy outcome; PPROM—premature preterm rupture of membranes; hDSC—human decidual stromal cell; TPL—threatened preterm labor; Ripk2—receptor-interacting protein kinase 2; NOD—nucleotide-binding oligomerization domain; T-antigen—Thomsen Friedenreich antigen; MsrAB—methionine sulfoxide reductase; Trx/CcdA—Thioredoxin/Cytochrome c.

**Table 2:** Summary of research articles and case reports linking *F. nucleatum* to various GDs.

SL. No.	Article Type	Country of Report	Year of Publication	Specific Findings/Main Highlights	References
1	Case report	UK	2019	*F. nucleatum-*induced peritonitis can complicate a clinical case of small hepatic vein” BCS in a symptomatic Pakistani woman.	Bannaga et al. [138]
2	Research article	USA	2020	*F. nucleatum* has a mutualistic relationship with the BV-associated bacteria such as *Gardnerella vaginalis* as they are major sialidase producers, enabling *F. nucleatum* to consume sialic acids from the host-produced mucus, thereby supporting colonization and vaginal dysbiosis.	Agarwal et al. [103]
3	Case report	Canada	2021	Association between *F. nucleatum* and a symptomatic case of chronic endometritis.	Mercer et al. [101]
4	Research article	USA	2022	The use of a multi-omics approach with 3-D cervical epithelial cell culture model reveals pro-inflammatory and metabolic changes (hallmarks of cancer) elicited by BV-associated *F. nucleatum*.	Maarsingh et al. [142]
5	Research article	India	2023	Significantly higher levels of *F. nucleatum* in the subgingival plaque samples in patients with PCOS and periodontitis and patients with PCOS and gingivitis, compared to the healthy individuals are indicative of the association between PCOS and oral microflora.	Achu Joseph et al. [31]
6	Research article	Japan	2023	Identification of a novel pathogenic mechanism of endometriosis involving *F. nucleatum* infection in the endometrium and its eradication by specific antibiotics against this bacterium that can serve as an attractive option for the treatment of endometriosis.	Muraoka et al. [30]

Abbreviations: GD—gynecological disease; BCS—Budd–Chiari syndrome; BV—bacterial vaginosis; PCOS—polycystic ovary syndrome.

**Table 3:** Summary of research articles and case reports linking *F. nucleatum* to various GCs.

SL. No.	Article Type	Country of Report	Year of Publication	Specific Findings/Main Highlights	References
1	Research article	Brazil	2019	*F. nucleatum*-induced chronic periodontitis, causing chronic inflammation may indirectly contribute to BC through different mechanisms.	Bernhard et al. [143]
2	Research article	Israel	2020	An analysis of more than 1000 tumor samples of seven cancer types, and adjacent noncancerous tissues, identifies tumor-type-specific microbiomes composed mostly of intracellular bacteria with *F. nucleatum* being concomitantly associated with BC.	Nejman et al. [182]
3	Research article	Israel	2020	High Gal-GalNAc level in BC tissues acts as an oncoantigen and plays a critical role by serving as a ligand to Fap2 adhesin of *F. nucleatum,* supporting colonization, promoting mammary tumor growth and progression and thereby indicating Fap2 as a potential drug target.	Parhi et al. [14]
4	Case series	Saudi Arabia	2020	*F. nucleatum*-associated bacteremia in a 72-year-old female patient with metastatic OC, dies within 30 days of detection, despite receiving 14 days of antibiotic treatment.	Almohaya et al. [144]
5	Research article	China	2020	Distinctively high levels of *F. nucleatum* in CC, especially in relapsed disease, CC cells with high burdens of *F. nucleatum* intratumoral infiltration exhibiting CSC characteristics and patients with high burdens of intratumorally infiltrated *F. nucleatum* displaying poor rates of both overall survival and PFS, thereby suggesting that *F. nucleatum* might be one potential CC diagnostic and prognostic biomarker.	Huang et al. [145]
6	Research article	India	2022	The use of a computational tool named IPD to identify infectious pathogens from heterogeneous NGS datasets does not reveal the enrichment of *F. nucleatum* in breast transcriptome samples.	Desai et al. [184]
7	Research article	Korea	2022	*F. nucleatum* infection in an OC patient, with isolates susceptible to all 10 antimicrobial agents tested.	Kim et al. [146]
8	Research article	Florida	2022	A significantly greater abundance of *F. nucleatum* in the vaginal samples of high-grade EC patients together with the non-significant increase in the low-grade EC patients, compared to those in benign individuals, suggest the bacterium’s role in tumor growth.	Hakimjavadi et al. [205]
9	Research article	USA	2022	The use of the multi-omics approach with 3-D cervical epithelial cell culture model reveals that *F. nucleatum* infection can promote HPV infection and persistence and consequently cervical neoplasia by generating pro-inflammatory responses and upregulating the metabolic hallmarks of CC.	Maarsingh et al. [142]
10	Research article	China	2023	*F. nucleatum*-derived small EVs can promote and enhance malignant manifestations of BC such as proliferation, migration, and invasion via TLR4.	Li et al. [179]
11	Research article	USA	2023	Enrichment of *F. nucleatum*, in patients with other OC histologies in comparison to the serous OC patients, within the whole OC cohort.	Asangba et al. [147]
12	Research article	Poland	2023	The vaginal and cervical microbiome of women with EC are enriched with *F. nucleatum* and this suggests that this bacterium is a potential endometrial cause/co-factor to promote/stimulate endometrial carcinogenesis.	Barczynski et al. [149]

Abbreviations: GC—gynecological cancer; BC—breast cancer; OC—ovarian cancer; EC—endometrial cancer; CC—cervical cancer; CSC—cancer stem cell; PFS—progression-free survival; IPD—infectious pathogen detector; NGS— next-generation sequencing; EV—extracellular vesicles; TLR4—Toll-like receptor 4.
